# Annotations

**Published:** 1888-11-10

**Authors:** 


### ANNOTATIONS.

"False Doctoring."

A WRITER in the *Cornhill* tells some amusing stories of the way in which some parts of the Prayer-book are misapprehended in certain outlying districts. It is not quite impossible that one or two of them might be found by a sceptical critic a little difficult of scientific verification. As stories, however, they are not without an element of fun. Among the rest is one of a certain Yorkshireman, who was much given to over-indulgence in quack medicines. The clergyman of his parish, who seems to have been soundly orthodox on the vital questions of pills and mixtures, grieved much over this case of medical schism. Finding remonstrance with the offender himself of no use, the clerical champion of medical orthodoxy appealed to the wife. He did not take the line of argument a doctor would have followed, and denounce the husband as a fool who wasted his money, and ruined his health in the process. He took the proper clerical ground. The swallowing of so much quack medicine "is a downright sin," said he. "I know it," replied the unhappy wife; "I know it; and many's the time I have prayed against it in the Church service." Here was a poser for the parish priest. In what part of the Prayer-book there might be found any collect or other petition against the too free use of quack medicines he was totally at a loss to think. There was nothing for it but to ask the woman; and she was quite ready with the answer. "From all false *doctoring*, . . . Good Lord deliver us." That was how she understood the word *doctrine;* and probably to her the sin of medical schism was much more practically harmful than that of ecclesiastical schism would have been. It is a pity to spoil the story by tacking on a moral; but as that is the purpose for which it is quoted, the moral must needs follow. It is this: That much physic from any source is usually injurious to health; but when it comes from the patent medicine vendor, its injuriousness reaches a maximum. The poorest doctor is better than the most plausible "quack."

The Flock or the Fleece?

IF a letter, signed "Nurses who are Writing from Experience," which appeared in the *Daily Chronicle* a short time ago, be a correct presentation of facts, there are gross scandals transpiring in the Hospital world. The nurses, in effect, say that many patients die in infectious hospitals without a word from priest or minister, even though the paid Chaplain has been early informed of their imminent danger. "Have you any grown-up patients dangerously ill in this ward?" says the Chaplain at the door. On being answered in the affirmative, he turns upon his heel with a "Very well, nurse, I will look in to-morrow." To-morrow the patient is probably dead. "In the children's ward particularly," say the nurses, "it would seem that age debars from receiving spiritual comfort." One case is given—that of a little girl eight years old, a bright and intelligent child. The Chaplain, when told that she was "very ill indeed," said: "Ah, she is too young to understand my preaching now; we will wait until she is better." The particular institution referred to was a fever hospital, and the "nurses" wish to know "why poor fever patients, who have so many long tedious hours to lie and think, and especially during convalescence, should not be benefited by the paid attention supposed to be received from the Chaplain." They point out that the other members of the hospital staff are seldom off duty, either on weekdays or Sundays; that they cannot get out to any place of worship; and that, notwithstanding this, no Sunday services of any kind are held for them, even though there is a paid Chaplain. Now, we do not know how far these "nurses" are stating facts without prejudice. If their story be at all correct, we venture to think that the particular Chaplain they have in view has made a serious mistake in taking the office he has, or even in being a clergyman at all. However it be, the matter should not be allowed to rest where it is. The nurses should give the names, both of Hospital and Chaplain, and the Metropolitan Asylums Board Managers should make an investigation. Paid ministers of religion are sometimes as essential as paid hospital matrons, but when the clergyman takes his stipend but fails to perform the duties of his office, or performs them perfunctorily, he is behaving as scandalously as the matron who should refuse to furnish the unhappy patients with food and medicine. We urge upon the nurses who have so far made the matter public, that they owe it to themselves and the Hospital world to fully declare all the facts, together with the names in question. Scandals of this kind are too shameful to be hid away in corners.

An Incompatible Mixture.

"ADVERSITY makes us acquainted with strange bedfellows." The condition of "adversity" into which most branches of the medical profession have now fallen has recently furnished an odd illustration of the truth of the old proverb. An audacious chemist, rendered bold by the successful sale of his wares, suddenly developes an ambition not uncommon among chemists. He resolves to be a "doctor." In the meantime, however, he has a good business in hand. The problem is how to retain it and make it dovetail into his new departure. The plan is simple: get a doctor to convert the drug business into a medical practice, and take advantage of the doctor's presence to attend a hospital and acquire a medical qualification. If the doctor manages well there will be a good practice at the end of the period of medical study for one or both of the partners. The thing looks eminently feasible. How to realise it in practice is the next question. Fortune favours the bold. Why not approach some medical man, and propose terms which shall be, at any rate, good for the proposer? This is done. The jaunty chemist makes his jaunty offer. The result is not without a dash of the comical. The doctor receives the offer with amazed indignation; with so much indignation, indeed, that he rushes incontinently into print. Like the outraged physician of Barchester, he will let the whole medical world know of this chemist's impudence; he will indeed. Meanwhile the chemist is probably laughing at him, and thinking that when he next approaches a medical man with a view to business he will make sure beforehand that the man of physic possesses a little common sense. When the matter is looked at without traditional spectacles it is at once apparent that the doctor who dispenses his own medicines carries on a business, one part of which is identical with a part of the chemist's. It is equally obvious that if a doctor and a chemist could work together in partnership there might, in some cases, be relief and profit to both. We do not counsel this. On the contrary, what we should advise would be that doctors give up dispensing medicines altogether, and that chemists give up prescribing altogether. That would be best for both. At present the doctors and chemists in all neighbourhoods, except the very wealthiest, are busily engaged in cutting each other's throats. The doctor cannot live by his fees because of the prescribing chemist in the next street; the chemist cannot live by his sales of drugs because of the dispensing doctor round the corner. The public may seem to profit, but does not, because the competition necessitates the lowering of the quality of the drugs and the diminishing of the time and care the doctor can afford for each patient. Absolute partnerships between doctors and chemists may be impracticable and undesirable; but reasonable mutual arrangements whereby each shall keep strictly to his own department would be an unmixed advantage both to doctors, chemists, and the public. Doctors and chemists are both soldiers in the same great army of healers, and it is as discreditable as it is dangerous for those who fight under the same flag to turn their weapons against each other in the face of the common enemy.

